# Recurrent upper limb ischemia caused by clavicular pseudarthrosis: an orthopedic cause of arterial thoracic outlet syndrome

**DOI:** 10.1016/j.xrrt.2026.100740

**Published:** 2026-04-01

**Authors:** Ignacio Pulido Cloquell, Jason Sepulle, Luis Carlos Silva Corten, Pierre Pirlot, Jean Schrooyen, Darius Lepot, Clement Stevens, Bruno Vincent

**Affiliations:** a Department of Orthopaedic Surgery, Clinique Saint-Pierre, Ottignies, Belgium; b Department of Vascular and Thoracic Surgery, Clinique Saint-Pierre, Ottignies, Belgium; c Department of Radiology, Cliniques Universitaires Saint-Luc, Brussels, Belgium

**Keywords:** Thoracic outlet syndrome, Clavicle fracture, Malunion, Pseudarthrosis, Upper limb ischemia, Case report

## Introduction

Arterial thoracic outlet syndrome (ATOS) is a rare entity, accounting for less than 5% of all TOS cases, and is most commonly associated with congenital anomalies or acquired post-traumatic deformities.^6^ Clavicular malunion or pseudarthrosis following nonoperative treatment of displaced midshaft fractures is a rare but recognized cause of subclavian arterial compression and ATOS.^2^^,^^5^ Symptoms may occur several years after the initial clavicular injury, leading to delayed diagnosis and exposing patients to thromboembolic and potentially limb-threatening ischemic complications.^3^^,^^5^

While ATOS is often managed from a vascular perspective, some cases arise from a purely orthopedic clavicular deformity. In such situations, correction of the clavicle rather than repeated vascular procedures constitutes the definitive treatment.^2^

## Case presentation

**Figure 1:**
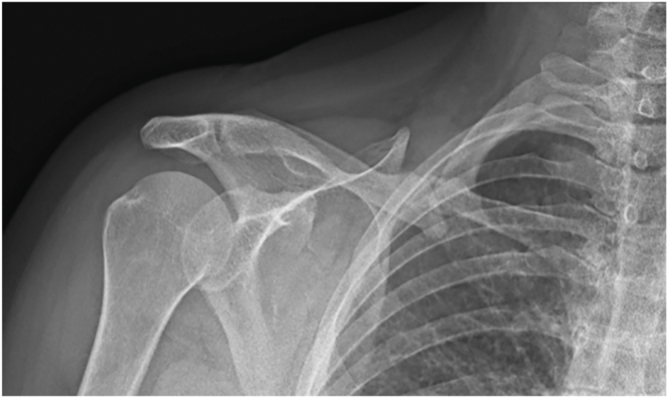
Frontal view radiograph showing a clavicular midshaft hypertrophic pseudarthrosis.

A 50-year-old woman was referred to the vascular surgery department for recurrent ischemic symptoms of the right upper limb. Her medical history included a right midshaft clavicle fracture sustained in 2010, initially managed nonoperatively with a figure-of-eight bandage. The fracture was consistent with a displaced AO 15.2A pattern (Fig. 1). The patient became asymptomatic after the initial treatment and was subsequently lost to follow-up.

### First ischemic episode

**Figure 2:**
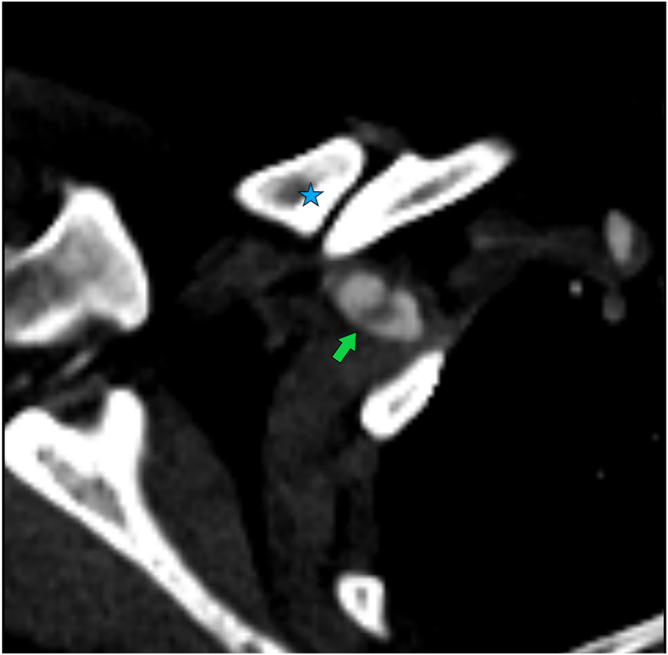
Axial CTA image showing, proximal to the thrombotic occlusion, an intimal flap inside the right subclavian artery (*greenarrow*) adjacent to the hypertrophic clavicular pseudarthrosis site (*blue star*).*CTA*, computed tomography angiography.

**Figure 3:**
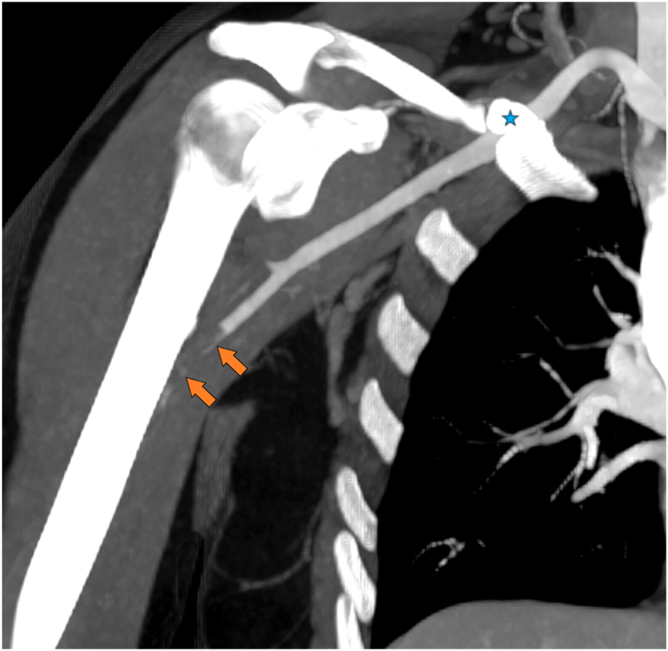
Coronal MIP CTA image showing complete thrombotic occlusion (*orangearrow*) at the axillohumeral arterial junction and, more proximally, a hypertrophic clavicular pseudarthrosis overlying the subclavian artery (*blue star*). *MIP*, Maximum Intensity Projection; *CTA*, computed tomography angiography.

In November 2023, the patient presented to the emergency department with pain, paresthesia, and pallor of the right upper limb. Clinical examination revealed asymmetric peripheral pulses. Computed tomography angiography (CTA) demonstrated a suspected intimal flap of the right subclavian artery adjacent to the previous clavicular fracture site, in the absence of visible arterial compression or direct contact (Fig. 2). More distally, a complete thrombotic occlusion was identified at the axillohumeral junction (Fig. 3).

On the same day, the patient underwent surgical Fogarty thrombectomy of the brachial, radial, and ulnar arteries. Completion arteriography showed no residual vascular abnormality.

Post-operative recovery was favorable, with progressive neurological improvement. Duplex ultrasound surveillance was reassuring, and follow-up CTA demonstrated no persistent vascular lesion. Anticoagulation therapy was discontinued one year later.

### Recurrence

One month after discontinuation of anticoagulation therapy, the patient presented again with pain, pallor, coolness, and paresthesia of the right upper limb. CTA revealed recurrent distal arterial occlusion with preserved proximal arterial patency, consistent with an embolic event. Repeat surgical thrombectomy followed by percutaneous transluminal angioplasty was performed, restoring distal arterial flow.

### Diagnostic workup

Detailed history-taking revealed that the patient habitually slept on her right side and experienced recurrent nocturnal and morning paresthesia of the right upper limb, suggesting positional clavicular hypermobility compatible with TOS.

**Figure 4:**
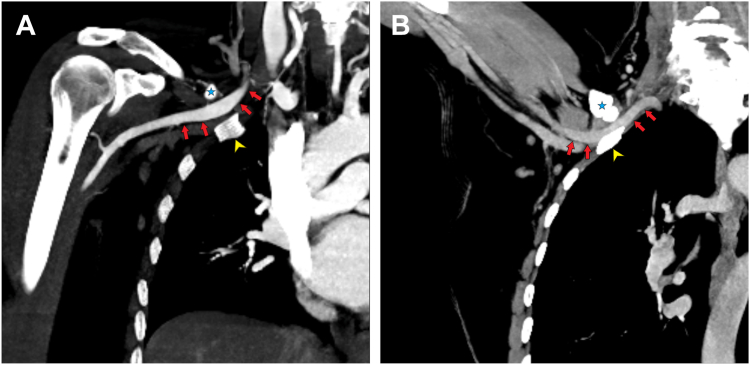
Coronal MIP CTA images showing normal arterial patency the arm adducted (**A**) and showing arterial compression the arm abducted/elevated (**B**) of the right subclavian artery (*redarrows*) between the hypertrophic clavicular pseudarthrosis (*blue star*) and first rib (*yellow arrowhead*). *MIP*, Maximum Intensity Projection; *CTA*, computed tomography angiography.

Duplex ultrasound demonstrated normal arterial flow at rest. However, positional ultrasound and CTA identified a hypertrophic clavicular pseudarthrosis causing dynamic compression of the subclavian vascular bundle, consistent with post-traumatic ATOS (Fig. 4).

### Definitive treatment

**Figure 5:**
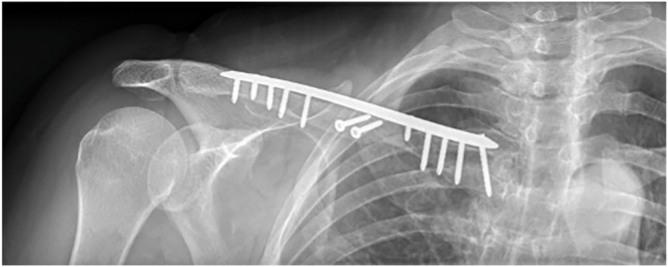
Frontal view post-operative radiograph showing correction of the hypertrophic callus and superior plate-and-screw osteosynthesis.

Given the confirmed mechanical orthopedic etiology, the patient underwent surgical débridement of the nonunion site followed by open reduction and internal fixation of the clavicular pseudarthrosis. Stabilization was achieved using 2 anteroposterior interfragmentary lag screws combined with a precontoured anatomic locking plate secured with 5 locking screws in each fragment (Fig. 5).

### Outcome and follow-up

Post-operatively, the patient regained full, symmetrical range of motion of both upper limbs. CT imaging performed in November 2025 showed that complete bony union had not yet been achieved; however, clavicular stability was satisfactory. Duplex ultrasound confirmed normal arterial flow without positional compression.

Following vascular reassessment in December 2025, anticoagulation therapy and neuropathic pain medication were discontinued. No recurrent ischemic or neurological symptoms were observed during follow-up.

## Discussion

Post-traumatic clavicular malunion or pseudarthrosis is a recognized but rare cause of ATOS.^2^^,^^5^^,^^7^ From an orthopedic perspective, this entity should be regarded as a late mechanical complication of clavicle fracture healing rather than a primary vascular disorder. Since the earliest descriptions, only a limited number of cases of TOS secondary to clavicular deformity have been reported, mostly as isolated case reports or small series, underscoring its rarity.^1^, ^2^, ^3^^,^^7^ The underlying mechanism involves abnormal clavicular mobility or hypertrophic callus formation producing dynamic compression of the subclavian artery, particularly during arm elevation or provocative maneuvers.^2^^,^^3^^,^^5^^,^^7^

Fujita et al^3^ reported one of the earliest well-documented cases of late-onset arterial TOS following conservative fracture treatment and emphasized the diagnostic value of dynamic imaging. Subsequent reports have described similarly delayed presentations, with latency periods ranging from months to decades after the initial trauma.^1^^,^^2^^,^^7^ These findings support the concept that progressive hypertrophic remodeling and repetitive mechanical irritation contribute substantially to vascular injury.^3^^,^^5^^,^^7^

Static imaging at rest frequently underestimates vascular compromise. Several authors have highlighted the importance of positional CTA to demonstrate arterial compression.^3^^,^^7^ In the present case, positional imaging proved decisive for diagnosis, whereas resting imaging and intraoperative angiography were initially reassuring.

Recurrent thromboembolic events are frequently reported when the underlying mechanical compression remains uncorrected. Although urgent vascular procedures such as thrombectomy or angioplasty are required in the setting of limb-threatening ischemia, they do not constitute definitive treatment. Most published cases demonstrate that correction of the clavicular deformity is essential to prevent recurrence.^1^^,^^2^^,^^5^^,^^7^

A distinctive feature of the present case is the occurrence of recurrent distal embolization originating from an intimal flap of the subclavian artery, likely induced by repetitive mechanical irritation from hypertrophic clavicular pseudarthrosis and compounded by intermittent positional compression during right-side sleeping. Whereas previous reports predominantly describe fixed stenosis, thrombosis, pseudoaneurysm formation, or extrinsic compression, distal embolization originating from an intimal flap secondary to clavicular pseudoarthrosis has not been previously reported.^2^^,^^5^^,^^7^ This observation suggests that abnormal clavicular mobility may contribute not only to dynamic arterial compression but also to repetitive arterial wall injury leading to intimal damage and distal embolic complications, with important implications for the long-term orthopedic follow-up of displaced clavicle fractures treated nonoperatively.^5^^,^^7^

Surgical correction of clavicular malunion or pseudarthrosis has been reported to relieve vascular compression and provide durable symptom resolution.^7^ In selected cases with associated costoclavicular narrowing, additional decompression procedures may be considered.^4^ However, when imaging clearly demonstrates an isolated clavicle-related mechanism, stabilization of the clavicle alone may be sufficient and can avoid the morbidity associated with more extensive decompression procedures.^1^^,^^2^^,^^7^

Finally, this case highlights that patients with displaced clavicle fractures treated nonoperatively may develop late and unexpected vascular complications, even decades after the initial injury.^3^^,^^7^ Long-term clinical vigilance and appropriate dynamic imaging should therefore be considered in symptomatic individuals.

## Review of published cases

Published reports of ATOS secondary to clavicular malunion or pseudarthrosis remain rare but are increasingly recognized as a cause of upper limb ischemia, distal embolization, and neurovascular compromise.^1^, ^2^, ^3^^,^^5^^,^^7^

Table I summarizes the principal cases available in the literature, focusing on patient demographics, delay from fracture to symptom onset, mechanism of vascular involvement, orthopedic and vascular management, and clinical outcomes. Overall, the literature highlights the heterogeneity of clinical presentations and collectively supports anatomical correction of the clavicular deformity as a key determinant of durable symptom resolution.

**Table I:** Key reported cases of arterial thoracic outlet syndrome secondary to clavicular malunion or pseudarthrosis.

Author (yr)	Age/sex	Delay from fracture	Vascular presentation	Orthopedic treatment	Outcome	Reference
Beliaev et al^1^	NR	Several yr	Arterial TOS	Clavicle deformity correction	Recovery	1
Connolly et al^2^	NR	Several yr	Arterial TOS	Corrective clavicle osteotomy	Symptom resolution	2
Fujita et al^3^	NR	Several yr	Late arterial TOS	Clavicle osteotomy	Symptom improvement	3
Furuhata et al^4^	46/F	21 yr	ATOS + neurologic symptoms	First rib resection + clavicle osteotomy	Resolution	4
Heyn et al^5^	NR	NR	Subclavian pseudoaneurysm/ATOS	Treatment of pseudarthrosis	Clinical improvement	5
Yoo et al^7^	NR	Variable	Arterial TOS	Corrective clavicle surgery	Symptom resolution	7
Present case	50/F	13 yr	Recurrent distal embolization	Débridement + plate fixation	No recurrence	—

*TOS*, thoracic outlet syndrome; *ATOS*, arterial thoracic outlet syndrome; *F*, female; *NR*, not reported.

## Conclusion

This case illustrates that clavicular pseudarthrosis may induce not only dynamic arterial compression but also intimal arterial injury leading to recurrent distal embolization. Post-traumatic clavicular deformity should therefore be recognized as a potential late mechanical cause of ATOS and recurrent limb ischemia, even many years after the initial fracture.

Positional CTA appears essential for diagnosis, particularly when resting imaging is inconclusive. When a clavicle-related mechanism is clearly identified, isolated surgical stabilization of the clavicle may provide definitive relief of vascular compression and durable prevention of further thromboembolic events, with sustained vascular and functional recovery.

## Disclaimers:

Funding: No external funding or grants were received for this study.

Conflicts of interest:The authors, their immediate families, and any research foundations with which they are affiliated have not received any financial payments or other benefits from any commercial entity related to the subject of this article.

Patient consent: Obtained.
